# Prognostic value of health‐related quality of life for death risk stratification in patients with unresectable glioblastoma

**DOI:** 10.1002/cam4.734

**Published:** 2016-06-01

**Authors:** Brice Paquette, Dewi Vernerey, Bruno Chauffert, Sandrine Dabakuyo, Loic Feuvret, Luc Taillandier, Didier Frappaz, Hervé Taillia, Roland Schott, François Ducray, Michel Fabbro, Isabelle Tennevet, François Ghiringhelli, Jean‐Sébastien Guillamo, Xavier Durando, Daniel Castera, Marc Frenay, Chantal Campello, Cécile Dalban, Jérome Skrzypski, Olivier Chinot, Amélie Anota, Franck Bonnetain

**Affiliations:** ^1^ Methodological and Quality of Life in Oncology UnitUniversity Hospital of BesançonBesançonEA 3181France; ^2^ Department of Digestive Surgery and Liver TransplantationUniversity Hospital of BesançonBesançonFrance; ^3^ Department of Medical OncologyUniversity HospitalEA 4666AmiensFrance; ^4^ Biostatistics and Quality of life UnitCentre Georges François LeclercDijonFrance; ^5^ Quality of Life in Oncology National PlatformBesançonFrance; ^6^ Department of RadiotherapyPitié‐Salpetrière University HospitalParisFrance; ^7^ Department of NeurologyUniversity HospitalNancyFrance; ^8^ Department of OncologyLeon Berard Centre for Fight against CancerLyonFrance; ^9^ Department of NeurologyHIA Val de GraceParisFrance; ^10^ Department of OncologyPaul Strauss Centre for Fight against CancerStrasbourgFrance; ^11^ Department of NeurologyUniversity HospitalLyonFrance; ^12^ Department of OncologyVal d'Aurelle Center for Fight against CancerMontpellierFrance; ^13^ Department of OncologyHenri Becquerel Center for Fight against CancerRouenFrance; ^14^ Department of OncologyGF Leclerc Center for Fight against CancerDijonFrance; ^15^ Department of NeurologyUniversity HospitalCaenFrance; ^16^ Department of OncologyJean Perrin Center for Fight against CancerClermont‐FerrandFrance; ^17^ Clinique Saint PierrePerpignanFrance; ^18^ Department of OncologyAntoine Lacassagne Center for Fight against CancerNiceFrance; ^19^ Department of NeurologyUniversity HospitalNimesFrance; ^20^ Methodology UnitGF Leclerc Center for Fight against CancerDijonFrance; ^21^ Department of Neuro‐OncologyUniversity Hospital La TimoneMarseilleFrance

**Keywords:** Health‐related quality of life, overall survival, prognostic score, risk stratification, unresectable glioblastoma

## Abstract

Glioblastoma is the most common malignant brain tumor in adults. Baseline health‐related quality of life (HRQoL) is a major subject of concern for these patients. We aimed to assess the independent prognostic value of HRQoL in unresectable glioblastoma (UGB) patients for death risk stratification. One hundred and thirty‐four patients with UGB were enrolled from the TEMAVIR trial. HRQoL was evaluated at baseline using the EORTC QLQ‐C30 and BN20 brain cancer module. Clinical and HRQoL parameters were evaluated in univariable and multivariable Cox analysis as prognostic factors for overall survival (OS). Performance assessment and internal validation of the final model were evaluated with Harrel's C‐index, calibration plot, and bootstrap sample procedure. Two OS independent predictors were identified: future uncertainty and sensitivity deficit. The final model exhibited good calibration and acceptable discrimination (C statistic = 0.63). The internal validity of the model was verified with robust uncertainties around the hazard ratio. The prognostic score identified three groups of patients with distinctly different risk profiles with median OS estimated at 16.2, 9.2, and 4.5 months. We demonstrated the additional prognostic value of HRQoL in UGB for death risk stratification and provided a score that may help to guide clinical management and stratification in future clinical trials.

## Introduction

Glioblastoma (GB), or WHO grade IV glioma, is the most common malignant brain tumor in adults, with an estimated incidence of between 1800 and 2400 cases per year in France 1. The incidence in Europe and North America is similar, at 2–3 per 100,000 adults per year 2. A minority of GBs are unresectable (UGB: RPA class V) 3. This disease is frequently revealed by a neurological deficit, whereas health status at diagnosis is mostly preserved 1. Nevertheless, the survival prognosis of patients with UGB remains extremely poor 1, 2.

In this context, health‐related quality of life (HRQoL) is a major subject of concern for patients with UGB, who are often symptomatic at the time of diagnosis and are confronted with cognitive deficit due to tumor burden 4, 5, 6, 7. In palliative care patients, the prognostic value of HRQoL has been demonstrated for several types of cancer 8. Although overall survival (OS) is still considered the “gold standard” for primary endpoints in oncology, most clinical trials now integrate HRQoL as one of the major key endpoints to investigate the clinical benefit of new therapeutic strategies for the patient. HRQoL is considered a valuable key endpoint by the American Society of Clinical Oncology and the Food and Drug Administration, which should be considered at least as a secondary endpoint, and if no effect on OS is observed could be considered as a primary or coprimary endpoint^9^, ^10^, ^11^. Thus, HRQoL could constitute relevant additional information along with conventional clinical and biological parameters for the improvement of death risk stratification in UGB patients.

The management of patients with UGB remains complex 12. Thus, there is a need for tools to optimize the selection of patients for different treatment options to achieve more personalized management. Specifically, better discrimination for predicting OS at diagnosis could be very useful for the stratification of various treatment options and to ensure well‐balanced arms in future clinical trials.

Consequently, we assessed the additional prognostic value of HRQoL in patients with UGB for death risk stratification among conventional parameters in a large phase II cohort 12 and propose a new prognostic score including HRQoL information.

## Patients and Methods

### TEMAVIR study

Individual patient data from the TEMAVIR phase II clinical trial were analyzed. The aim of this French multicenter study was to evaluate bevacizumab (BEV) and irinotecan (IRI) as neoadjuvant and adjuvant treatment combined with temozolomide (TMZ)‐based chemoradiation in UGB patients. The study has been extensively described elsewhere 12.

The inclusion criteria were as follows: patients with de novo unresectable supratentorial glioblastoma, histologically confirmed, with Karnofsky performance status over 50%. Only UGB patients were included. A urine protein test had to be negative, and systolic blood pressure had to be less than 170 mmHg.

The exclusion criteria were as follows: cardiovascular contraindication to BEV, anticoagulant or antiaggregant treatment, history of digestive hemorrhage and/or gastroduodenal ulcer, and brain hemorrhage at the initial MRI.

All patients were fully informed of the study and provided signed written informed consent. The trial was approved by the East France Ethics Committee no. 1 and was registered under EUDRACT number 2008‐002775‐28 (NCT01022918).

Patients were randomly (ratio 1:1) assigned to receive BEV and IRI as a neoadjuvant and adjuvant treatment combined with TMZ or only TMZ as neoadjuvant and adjuvant treatment. Stratification based on a minimization procedure was conducted according to Mini Mental State Examination 13 (MMSE <27 vs. ≥27), MRC neurological status (0, 1, 2 vs. 3, 4), gender, age (<50 vs. ≥50), and center.

The results and methodology of this trial have been presented extensively elsewhere 12.

### Baseline evaluation and parameters

The patient's clinical parameters at enrollment were collected using a demographic form (gender, age, treatment arm, Karnofsky performance status (0: worst to 100: best, 10 by 10) with the addition of a large spectrum for neurological status assessment (MMSE: 0–30; neurological status (0 = worst to 4 = best in increments of 1), headache (Yes/No), motor deficit (Yes/No), cognitive impairment (Yes/No), seizures (Yes/No), sensory deficit (Yes/No), sensitivity (cutaneous) deficit (Yes/No)).

### Health‐related quality‐of‐life assessment

HRQoL was evaluated using the European Organization for Research and Treatment of Cancer (EORTC) QLQ‐C30 cancer‐specific questionnaire 14 and its BN20 brain cancer‐specific module 15 at inclusion. When necessary (e.g., in the case of cognitive impairment), the completion of the questionnaires could be performed with the assistance of the Clinical Research Assistant involved in the study.

The QLQ‐C30 includes 30 items and measures five functional scales (physical, role, emotional, cognitive, and social functioning), global health status (GHS), financial difficulties and eight symptom scales (fatigue, nausea and vomiting, pain, dyspnea, insomnia, appetite loss, constipation, and diarrhea) 16.

The QLQ‐BN20 brain cancer‐specific module includes four symptoms scales and seven single items (future uncertainty, visual disorder, motor dysfunction, communication deficit, headache, seizures, drowsiness, itchy skin, hair loss, weakness of legs, and bladder control) 16.

These scores vary from 0 (worst) to 100 (best) for the functional dimensions and GHS and from 0 (best) to 100 (worst) for the symptom dimensions and were generated according to the EORTC Scoring Manual 16.

### Statistical analysis

All randomized patients with available HRQoL data at baseline were included in the analysis, whatever the respect of the eligibility criteria (modified intent‐to‐treat population).

The baseline characteristics of patients with or without HRQoL data were described by mean (SD) and frequency (percentages) for the continuous and categorical variables, respectively. The means and the proportions were compared using Student's *t*‐test and the chi‐squared test (or Fisher's exact test, if appropriate), respectively.

OS was defined as the time from randomization to death from any cause. Alive patients were censored at the last follow‐up or at the end of the study. OS was estimated using the Kaplan–Meier method and described using median or rate at specific time points with a 95% confidence interval (CI). Follow‐up was calculated using reverse Kaplan–Meier estimation 17.

Univariable and multivariable analyses were performed using Cox proportional hazards models, with estimation of the hazard ratio (HR) and the corresponding 95% two‐sided confidence interval (CI). Hazard proportionality was checked by plotting log‐minus‐log survival curves.

The association of clinical and HRQoL factors (QLQ‐C30 and QLQ‐BN20 supplementary module) with OS was first assessed in univariable analyses. HRQoL scores were dichotomized according to their observed statistical distributions (0 vs. > 0 or <50 vs. ≥50) or kept as continuous variables when possible, that is, when an approximately normal distribution was observed (particularly for dimensions evaluated by at least three items). For dimensions evaluated by one item, a dichotomization of <50 vs. ≥50 corresponds to (0; 33.3) vs. (66.7; 100).The correlation between HRQoL scores was controlled by evaluating the Pearson correlation coefficient to avoid colinearity. A multivariable analysis for HRQoL factors only was then performed. All variables with a *P*‐value <0.1 in univariable analysis were included in a stepwise backward elimination procedure to identify and select the HRQoL parameters associated with OS. The same procedure was then performed for biological and tumoral parameters.

The clinical factors identified in the two multivariable analysis of (1) HRQoL, (2) biological and tumoral parameters were thereafter included in a final multivariable model. Concato rules 18 were assumed for multivariable Cox models (1 variable per 10 events).

The accuracy of the final multivariable model was checked regarding two parameters: discrimination and calibration. The predictive value and the discrimination ability (i.e., the ability to separate patients with different prognoses) of the model were evaluated with Harrell's Concordance (C)‐index. One thousand random samples from the population were used to derive the 95% CI for the Harrell's Concordance statistic. The C‐index estimates the proportion of all pairwise patients' combinations from the sample data whose survival time can be ordered such that the patient with the highest predicted survival is the one who actually survived longest (discrimination). The C‐index (0 ≤ *C* ≤ 1) is a probability of concordance between predicted and observed survival, with *C* = 0.5 for random predictions and *C* = 1 for a perfectly discriminating model. The calibration and goodness of fit of the model were assessed using a calibration plot. Calibration refers to the ability to provide unbiased survival predictions in groups of similar patients. A predictive model for death was considered “well calibrated” if the difference between predictions and observations for death in all groups of similar patients was close to 0 (perfect calibration). Any large deviation indicated a lack of calibration.

The internal validity of the model was assessed using a bootstrap sample procedure. Several approaches have been proposed to assess the performance in samples of the same population (internal validation). Bootstrapping is the preferred simulation technique and was first described by Bradley Efron 19. The idea is that the original dataset is a random sample of patients, representative of a general population. Bootstrapping involves generating a large number of datasets, each with the same sample size as the original one, by resampling with replacement (i.e., an already selected patient may be selected again).

We further focused on the improvement in model performance because of the inclusion of HRQoL parameters comparing two sets of predictions of OS probability: one set of predictions based on a Cox proportional hazards model without HRQoL parameters (including only independent clinical predictors for OS) and one set with HRQoL parameters. The discrimination ability and incremental value of HRQoL parameters were evaluated by *C* statistics. This analysis was repeated 1000 times using bootstrap samples to derive 95% CIs for the difference in the *C* statistic between models.

Lastly, for clinical practice, we investigated the possibility of providing a simple score based on the final multivariable model with the determination of cut‐off values for the continuous factors selected. Characteristics of population with distinctly different risk profiles identified with the score were then provided.

The analyses were conducted using SAS 9.3 (Statistical Analysis System, Cary, NC) and R 3.1.0 20. All statistical tests were two‐sided, and probability values <0.05 were regarded as significant.

## Results

### Overall patients' characteristics and HRQoL availability

In total, 134 patients were randomized in the study from April 2009 to January 2011 (67 in each treatment arm). HRQoL data were available for 102 (76.1%) of these patients.

The baseline characteristics of all patients and HRQoL availability are summarized in Table 1.

**Table 1:** Baseline characteristics of patients according to HRQoL availability

	All patients	Available HRQoL	Missing HRQoL	*P*
	(*N* = 134)	(*N* = 102)	(*N* = 32)	
	*N*(%)	*N*(%)	*N*(%)	
Age				
<50	17 (13.0)	9 (8.8)	8 (25.0)	
>50	117 (87.0)	93 (91.2)	24 (75.0)	**0.03**
Gender				
Male	80 (59.7)	60 (58.8	20 (59.4)	
Female	54 (40.3)	42 (41.2	12 (40.6)	0.9
Karnofsky performance status				
50%	13 (9.7)	10 (9.8)	3 (9.4)	
60%	16 (12.0)	12 (11.8)	4 (12.5)	
70%	33 (24.6)	25 (24.5)	8 (25.0)	
80%	32 (23.9)	25 (24.5)	7 (21.9)	
90%	26 (19.4)	18 (17.6)	8 (25.0)	
100%	13 (9.7)	11 (10.8)	2 (6.1)	
NA	1 (0.7)	1 (1.0)	0 (0.0)	0.9
Mini mental state examination				
<27	65 (48.5)	44 (43.1)	21 (65.6)	
>27	69 (51.5)	58 (56.9)	11 (34.4)	**0.04**
Neurological status				
SN0	9 (6.7)	9 (8.8)	0 (0.0)	
SN1	44 (32.8)	36 (35.3)	8 (25.0)	
SN2	39 (29.1)	28 (27.4)	11 (34.4)	
SN3	39 (29.1)	27 (26.5)	12 (37.5)	
SN4	3 (2.3)	2 (2.0)	1 (3.1)	0.3
Treatment arm				
A	67 (50.0)	52 (51.0)	15 (46.9)	
B	67 (50.0)	50 (49.0)	17 (53.1)	0.8
*Symptoms*				
Headache				
No	103 (76.9)	79 (77.4)	24 (75.0)	
Yes	30 (22.4)	22 (21.6)	8 (25.0)	
NA	1 (0.7)	1 (1.0)	0 (0.0)	0.9
Motor deficit				
No	73 (54.5)	58 (56.9)	15 (46.9)	
Yes	58 (43.3)	41 (40.2)	17 (53.1)	
NA	3 (2.2)	3 (2.9)	0 (0.0)	0.3
Cognitive impairment				
No	71 (53.0)	62 (60.8)	9 (28.1)	
Yes	62 (46.2)	39 (38.2)	23 (71.9)	
NA	1 (0.8)	1 (1.0)	0 (0.0)	0.002
Seizures				
No	124 (92.5)	93 (91.2)	31 (96.9)	
Yes	7 (5.2)	6 (5.8)	1 (3.1)	
NA	3 (2.3)	3 (3.0)	0 (0.0)	0.9
Sensory deficit				
No	110 (82.0)	86 (84.3)	24 (75.0)	
Yes	21 (15.7)	13 (12.7)	8 (25.0)	
NA	3 (2.3)	3 (3.0)	0 (0.0)	0.2
Sensitivity deficit				
No	108 (80.6)	84 (82.3)	24 (75.0)	
Yes	24 (17.9)	16 (15.7)	8 (25.0)	
NA	21.5	2 (2.0)	0 (0.0)	0.4
Total number of deaths	110	83	27	0.9
Median follow‐up (months)	24.0	24.0	23.1	0.67

The mean age of all randomized patients was 59.6 (SD = 7.0), with 117 patients (87%) over 50. Eighty patients (59.7%) were males. According to the classification RPA, 87% were RPA V.

There were 65 patients (48.5%) with an MMSE score under 27. The distribution of Karnofsky performance status scores and MRC neurological status are described in Table 1.

No significant differences were found in terms of gender, Karnofsky performance status scores or MRC neurological status between patients with or without HRQoL data. However, the MMSE score was significantly more frequently under 27 in patients with missing HRQoL data (43.1% vs. 65.6%, *P *= 0.004). Patients with HRQoL data were also significantly older than 50 (91.2% vs. 75%, *P *= 0.03).

Symptoms at baseline (i.e., headache, motor deficit, cognitive impairment, seizures, sensory deficit, and sensitivity deficit) are described in Table 1. Cognitive impairment was significantly more frequently observed in patients with missing HRQoL data (38.2% (*n* = 39) vs. 71.9% (*n* = 23), *P *= 0.002).

The median follow‐up was 24 months (95% CI) in patients with available HRQoL data versus 23.1 months (95% CI) in patients with missing HRQoL data (*P* = 0.67).

The median OS was 10.2 months (95% CI: 8.6–15.6) and 11.6 months (95% CI: 8.8–18.5) for patients with and without HRQoL data, respectively. No significant OS difference was found between these two groups (log‐rank *P *=* *0.86).

### Distribution of health‐related quality‐of‐life dimensions

The distributions of QLQ‐C30 and supplementary module BN20 scores are described in supplementary Table S1. All functional dimension scores presented a median greater than 50. Conversely, symptom dimension scores presented a median lower than 33.

Interestingly, we observed some variability in HRQoL parameters (e.g., median score equal to 50, 33.3, and 41.7 for global health status, fatigue, and future uncertainty, respectively) reflecting the potential relevance of this information for the improvement in death risk stratification, since the median is not equal to 0 or 100.

### Future uncertainty and sensitivity deficit are two independent prognostic factors for OS

The associations of clinical and HRQoL (QLQ‐C30 and QLQ‐BN20) parameters with risk of death for univariable and multivariable analysis are shown in Tables 2 and 3.

**Table 2:** Univariable analyses of clinical and HRQoL parameters

	*N*	Events	Hazard ratio	95% CI	*P*‐value
Biological parameters					
Hemoglobin^c^	96	79	1.08	0.92–1.27	0.36
White blood cell^c^	102	82	1	0.99–1.00	0.7
PNN^c^	102	82	1	1.00–1.00	0.13
Platelets^c^	102	82	1	1.00–1.00	**0.03**
Prothrombin rate^c^	82	66	1.01	0.98–1.05	0.48
Creatinemia^c^	101	82	1.01	1.00–1.03	**0.03**
Total Bilirubin^c^	95	77	1.01	0.96–1.06	0.70
AST^c^	99	80	1.02	0.98–1.06	0.25
ALT^c^	99	80	1.01	0.99–1.02	**0.06**
Clinical parameters					
Age^c^	102	82	1.02	0.99–1.06	0.169
Sex (women)	102	82	0.78	0.50–1.22	0.276
Karnofsky performance status	101	81	0.91	0.79–1.05	0.208
MMSE (>27)	102	82	0.69	0.44–1.08	0.099
Neurological status (>2)	102	82	1.80	1.15–2.82	**0.009**
Treatment arm (Bevacizumab – Irinotecan)	102	82	0.99	0.64–1.53	0.966
Headache/intracranial hypertension	101	81	1.25	0.73–2.14	0.423
Motor deficit	99	79	1.30	0.83–2.05	0.254
Cognitive impairment/behavior disorder	101	81	1.16	0.74–1.82	0.509
Seizures	99	79	0.38	0.12–1.23	0.107
Sensory deficit	99	79	1.74	0.96–3.18	0.069
Sensitivity deficit	100	80	2.84	1.58–5.13	**0.0005**
*Tumor characteristics*					
Laterality					
Bilateral	102	82	3.7288	0.47–29.25	
Right location	102	82	2.1267	0.29–15.62	
Left location	102	82	2.9277	0.40– 21.36	0.25
Location					
Frontal	102	82	1.14	0.73–1.77	0.57
Parietal	101	81	0.77	0.47–1.24	0.28
Temporal	101	81	0.88	0.56–1.38	0.57
Occipital	101	81	0.63	0.25–1.58	0.33
Thalamic	101	81	0.88	0.12–6.39	0.90
Other location	102	82	1.78	1.11–2.83	**0.01**
*Health‐related quality‐of‐life parameters*					
QLQ‐C30 scores					
Global health status^c^	98	79	0.99	0.98–1.01	0.201
Physical functioning^c^	98	79	0.99	0.98–1.01	0.368
Role functioning^c^	98	79	0.99	0.99–1.00	0.132
Emotional functioning^c^	98	79	0.99	0.98–1.00	0.059
Cognitive functioning^c^	99	80	0.99	0.99–1.01	0.507
Social functioning^c^	97	79	0.99	0.99–1.00	0.169
Fatigue^c^	100	81	1.00	0.99–1.01	0.700
Nausea and vomiting^b0^	100	81	1.00	0.99–1.02	0.497
Pain^b0^	99	80	1.01	0.99–1.01	0.079
Dyspnea^b0^	98	79	1.00	0.99–1.01	0.426
Insomnia^b50^	95	78	1.00	0.99–1.01	0.849
Appetite loss^b0^	96	78	1.00	0.99–1.01	0.842
Constipation^b0^	97	78	1.00	0.99–1.01	0.250
Diarrhea^b0^	99	80	1.01	0.99–1.02	0.115
Financial difficulties^b0^	96	77	1.02	1.01–1.03	**0.0007**
QLQ‐BN20 scores					
Future uncertainty^c^	99	80	1.01	0.99–1.02	**0.007**
Visual disorder^b50^	97	78	1.01	1.00–1.02	**0.037**
Motor dysfunction^b50^	98	79	1.00	0.99–1.01	0.324
Communication deficit^b50^	99	80	1.00	0.99–1.01	0.555
Headache^b50^	99	80	1.01	0.99–1.01	0.110
Seizures^b0^	94	77	1.01	0.99–1.02	0.484
Drowsiness^b50^	99	80	1.00	1.00–1.01	0.335
Itchy skin^b0^	96	77	1.01	0.99–1.02	0.075
Hair loss^b0^	88	70	0.97	0.93–1.02	0.231
Weakness of legs^b50^	96	77	1.00	0.99–1.01	0.905
Bladder control^b0^	97	78	1.00	0.99–1.01	0.803

Dichotomization: b0 = 0 versus > 0 or b50 = <50 versus > 50.

CI, confidence limits; c, continuous variable.

**Table 3:** Multivariable analyses

		*N*	Events	HR	95% CI	*P*‐value
A. Stepwise multivariable model for HRQoL parameters (*N* = 91)						
Future uncertainty^c^		91	72	1.010	1.002–1.018	**0.0138**
Financial difficulties		72	54	1		
		19	18	2.095	1.167–3.762	**0.0132**
B. Stepwise multivariable model for biological parameters (*N* = 74)						
Platelets		74	63	1.000	1.000–1.000	**0.0053**
Creatinemia		74	63	1.018	1.001–1.035	**0.0339**
C. Final full multivariable model (*N* = 91)						
MMSE <27	<27	38	34	1		
>27	>27	53	39	0.767	0.416–1.414	0.3954
SN ≤2	≤2	43	31	1		
>2	>2	48	42	1.125	0.556–2.277	0.7426
Sensory deficit	No	80	62	1		
	Yes	11	11	1.583	0.783–3.19	0.2009
Sensitivity deficit	No	78	61	1		
	Yes	13	12	2.416	1.088–5.369	**0.0303**
Future uncertainty^c^		91	73	1.012	1.003–1.020	**0.0091**
D. Final multivariable model (*N* = 97)						
Future uncertainty^c^		97	78	1.011	1.004–1.019	0.0040
Sensitivity deficit		97	78	2.828	1.539–5.197	0.0008

c, continuous variable.

In the univariable analysis, 13 variables were identified as prognostic factors for OS with a *P* < 0.1: platelet count (HR = 1, 95% CI: 1.00–1.00; *P *=* *0.03), creatinemia (HR = 1.01, 95% CI: 1.00–1.03, *P *=* *0.03), ALT (HR = 1.01, 95% CI: 0.99– 1.02, *P *=* *0.06), MMSE (HR = 0.69, 95% CI: 0.44–1.08, *P *=* *0.099), neurological status (HR = 1.80, 95% CI: 1.15– 2.82, *P *=* *0.009), sensory deficit (HR = 1.74, 95% CI: 0.96– 3.18, *P *=* *0.069), sensitivity deficit (HR = 2.84, 95% CI: 1.58–5.13, *P *<* *0.001), emotional functioning (HR = 0.99, CI: 0.98–1.00, *P *=* *0.059), pain (HR = 1.01, 95% CI: 0.99–1.01, *P* = 0.08), itchy skin (HR = 1.01, CI: 0.99–1.02, *P *=* *0.075), financial difficulties (HR = 1.02, 95% CI: 1.01–1.03, *P *<* *0.01), future uncertainty (HR = 1.01, 95% CI: 0.99–1.02, *P *<* *0.01), and visual disorder (HR = 1.01, 95% CI: 1.00–1.02, *P *=* *0.037).

Of note, other location that conventional (frontal, temporal, parietal, occipital, or thalamic) was found to be associated with OS (*P *=* *0.01). This information has to be handled with care due to the multiplicity of location by patient (47, 43, 11, and 1 patients have 1, 2, 3, or 4 locations, respectively).

Then, in a multivariable analysis for the HRQoL factors block, only financial difficulties (HR = 1.83, 95% CI: 1.09–3.087, *P *=* *0.02) and future uncertainty dimensions (HR = 1.01, 95% CI: 1.001–1.017], *P *=* *0.02) remained significantly independently associated with OS (Table 3). No colinearity between HRQoL scores incorporated in the multivariate analysis was reported (correlation coefficient was lower than 0.4 for each comparison).

Finally, when considering the clinical factors with a *P *<* *0.1 highlighted in the univariable analysis with the two HRQoL factors previously identified in the HRQoL factors block multivariable analysis, only two independent predictors were significantly associated with OS in the final multivariable analysis: future uncertainty score (HR = 1.01, 95% CI: 1.00–1.02, *P *=* *0.005) and presence of sensitivity deficit (HR = 2.77, 95% CI: 1.52–5.09, *P *=* *0.005) (Table 3).

### Final multivariable model performance assessment

**Figure 1:**
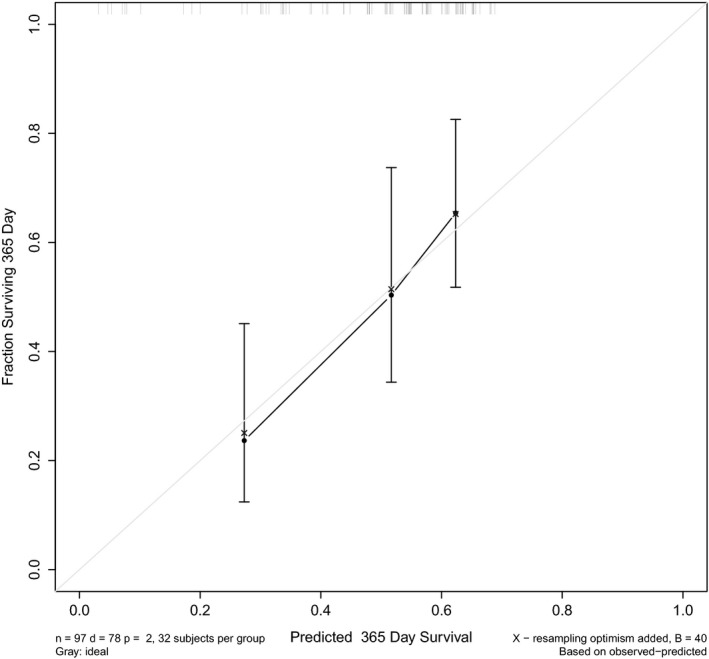
Calibration curve for the OS prediction OS at 1 year according to the final multivariable model. Final multivariable model‐predicted probability of overall survival is plotted on the *x*‐axis; actual overall survival is plotted on the *y*‐axis.

Our final multivariable Cox model exhibited acceptable discrimination (*C* statistic 0.63 [0.56–0.71]) and a good calibration, as shown in the calibration plot (Fig. 1), with an optimal agreement between the model prediction and actual observation.

### Internal validation of the final model

With the replicated datasets (*N* = 1000) derived from the bootstrap sample procedure, uncertainties around HR estimates can be measured.

Bootstrapping results for the internal validation reflect the robustness of the final model (HR 95% CI percentile: 1.002–1.020 and 1.710–4.742 for future uncertainty score and sensitivity deficit, respectively).

### Additional value of future uncertainty for OS prediction

The inclusion of the future uncertainty parameter in the reference model (including only sensitivity deficit) was found to significantly improve the discriminative ability of the model because the *C* statistic increased significantly from 0.56 to 0.63 (bootstrap mean difference = 0.07; 95% CI: 0.01–0.13). These results show that the addition of the future uncertainty (i.e., HRQoL information) to clinical parameters improved the stratification of patients at risk for death.

### Prognostic survival in unresectable glioblastoma patients (PROSUG) score

After the statistical investigation and determination of the importance for baseline prediction of future uncertainty and sensitivity which are key parameters in the prediction of OS, we explored the possibility to provide a simple score based on this multivariable model in clinical practice.

### Cut‐off value of future uncertainty fixed at 50 points

Simple implementation of future uncertainty monitoring in clinical practice is first guided by the determination of a relevant cut‐off to categorize patients into groups with low and high future uncertainty level at baseline.

The future uncertainty is a score on a 0–100 scale, and the median value in our study population was equal to 41.7 for the 102 patients included in the final analysis. Thus, a level of 50 seemed to be a relevant choice for a cut‐off value in clinical practice.

Considering the future uncertainty cut‐off value equal to 50, we investigated the interest in a combination of future uncertainty simple binary information and sensitivity information for the prediction of OS in clinical practice.

### Kaplan–Meier curves for OS according to future uncertainty and sensitivity parameters

**Figure 2:**
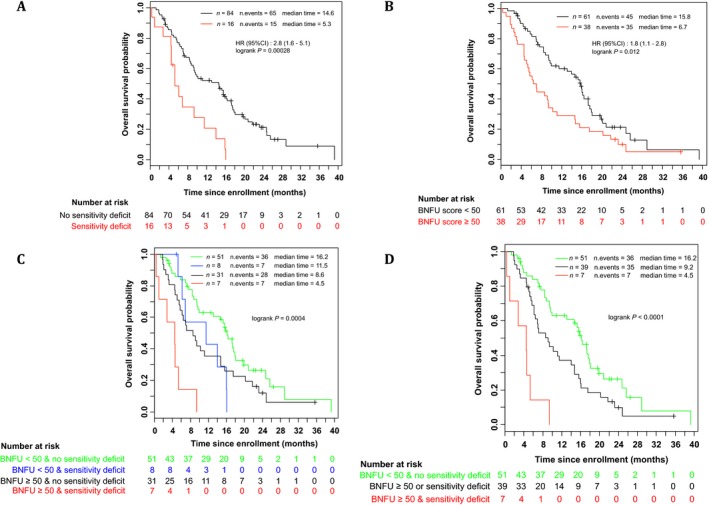
Kaplan–Meier curve of OS according to BNFU score (<50 or >50) (Panel A), sensitivity deficit status (presence or absence) (Panel B), Kaplan–Meier curves of OS according to BNFU score (<50 or >50) (Panel C), and sensory deficit status (presence or absence) (Panel D).

First, the median OS was significantly better in patients with a lower future uncertainty score than in patients with a high level of future uncertainty (median OS = 15.8 months (95% CI: 11.5–17.6) vs. 6.7 months (95% CI: 5.1–11.1), respectively, *P *= 0.011) (Fig. 2, panel A). Then, the median OS was significantly better in patients without sensitivity deficit than in patients with sensitivity deficit (median OS = 14.6 months (95% CI: 9.4–17.3) versus 5.3 months (95% CI: 4.5–14), respectively, *P* < 0.01) (Fig. 2, panel B).

Next, when combining future uncertainty and sensitivity categorical information, we were able to determine four subgroups of patients:

1. patients with a future uncertainty score <50 without sensitivity deficit (*N* = 51, 52.6%),
2. patients with a future uncertainty score <50 with sensitivity deficit (*N* = 8, 8.2%),
3. patients with a future uncertainty score >50 without sensitivity deficit (*N* = 31, 32%),
4. patients with a future uncertainty score >50 with sensitivity deficit (*N* = 7, 7.2%), (Fig. 2, panel C).

Considering the similar intermediate‐risk profile for the second and third group, these groups were pooled.

Finally, three groups of patients were identified with distinctly different risk profiles (Fig. 2, panel D), leading to the proposed PROSUG score (Supplementary table S2):

A low‐risk group: patients with a future uncertainty score <50 without sensitivity deficit (*N* = 51, 52.6%, median OS = 16.2 months, 95% CI: 13.0–19.8);
an intermediate‐risk group: patients with a future uncertainty score >50 or with sensitivity deficit, (*N* = 39, 40.2%, median OS = 9.2 months, 95% CI: 6.4–14.7); and
a high‐risk group: patients with a future uncertainty score >50 with sensitivity deficit (*N* = 7 7.2%, OS = 4.5 months, 95% CI: 1.0–NA),

with a global *P*‐value for log‐rank test <0.001.

### Characteristics of patients according to PROSUG score risk profile

Among the 97 patients (72.3%) involved in the final multivariable analysis, 51 (52.6%) were in the low‐risk group, 39 (40.2%) were in the intermediate group and 7 (7.2%) were in the high‐risk group. As described in Table 4, no significant differences were found among the three groups in term of age, treatment arm, headache, motor deficit, cognitive impairment, seizures, or sensory deficit. More women and patients presenting a lower Karnofsky performance status were in the high‐risk group (*P *=* *0.03 and *P *=* *0.05, respectively). Considering neurocognitive parameters, MMSE scores were significantly lower (<27) and neurological status significantly higher in the high‐risk group (*P *<* *0.01 in both cases). Future perspective score was significantly higher and sensitivity deficit was significantly more frequent in the high‐risk group (*P *<* *0.01 in both cases).

**Table 4:** Baseline characteristics according to risk level

	Low (*N* = 51)		Intermediate (*N* = 39)		High (*N* = 7)		*P*‐value
	*N*	%	*N*	%	*N*	%	
Age. mean (SD)	60.0 (6.2)		59.1 (7.2)		61.6 (7.5)		0.57
Gender							
Male	29	56.9	27	69.2	1	14.3	
Female	22	43.1	12	30.8	6	85.7	0.03
Karnofsky performance status							
50%	2	3.9	7	18.4	1	5.9	
60%	7	13.8	4	10.5	1	5.9	
70%	7	13.8	11	28.9	4	82.3	
80%	14	27.4	10	26.3	1	5.9	
90%	12	23.5	4	10.5	0	0.0	
100%	9	17.6	2	5.3	0	0.0	0.05
MMSE							
<27	15	29.4	23	59.0	4	57.1	
>27	36	70.6	16	41.0	3	42.9	<0.01
Neurological status							
SN0	7	13.7	2	5.1	0	0.0	
SN1	28	54.9	7	17.9	0	0.0	
SN2	6	11.8	16	41.0	2	28.6	
SN3	10	19.6	12	30.8	5	71.4	
SN4	0	0.0	2	5.1	0	0.0	<0.01
Treatment arm							
A	24	47.0	23	59.0	3	42.9	
B	27	53.0	16	41.0	4	57.1	0.53
*Symptoms*							
Headache							
Yes	13	25.5	6	15.4	2	28.6	
No	38	74.5	33	84.6	5	71.4	0.47
Motor deficit							
Yes	17	33.3	17	43.6	5	71.4	
No	34	66.7	21	56.4	2	28.6	0.12
Cognitive impairment							
Yes	20	39.2	14	35.9	1	14.3	
No	31	60.8	25	64.1	6	85.7	0.54
Seizures							
Yes	4	7.8	2	5.1	0	0.0	
No	47	92.2	36	94.9	7	100.0	0.68
Sensory deficit							
Yes	3	5.9	8	20.5	1	14.3	
No	48	94.1	30	79.5	6	85.7	0.08
Sensitivity deficit							
Yes	0	0.0	8	20.5	7	100.0	
No	51	100	31	79.5	0	0.0	<0.01
BNFU	24.3	13.6	59.6	26.4	83.3	11.8	<0.01

## Discussion

This study is the first to explore the prognostic value of baseline HRQoL in UGB patients, for whom survival prognosis is clearly worse than patients with resectable tumors 21.

Self‐reported HRQoL is known to be associated with OS in several types of cancer 22, 23. In this study, two independent key predictors for OS were identified in the final multivariable analysis: an HRQoL parameter, the future uncertainty dimension from the QLQ‐BN20 questionnaire, and a clinical variable, the sensitivity deficit status. Even if the financial difficulties dimension was eliminated after stepwise backward elimination, Minaya et al. described this trend when the QLQ‐BN20 HRQoL questionnaire was applied to caregivers^24^. Worries about financial issues may be specific to patients with brain tumors, even with a high protection‐level health system. Moreover, financial worries were in line with the prognostic value of future uncertainty. Therefore, these dimensions may reflect a sense of fragility among these patients regarding the future and may explain their impact on OS prediction.

These findings provide the opportunity for the construction of a simple score combining these independent predictors for OS in patients with UGB. This score identifies three subgroups of patients with distinctly different prognostic profile: low‐, intermediate‐, and high‐risk of death groups. This prognostic score could help to improve the classification of patients into risk populations and to be more precise in the assignment of patients to a specific therapeutic strategy.

Interestingly, the future uncertainty dimension from the QLQ‐BN20 questionnaire was used but not highlighted in the final model in the EORTC study of Mauer et al 4. Nevertheless, the previous study focused not only on patients with UGB but also on those with resectable tumors, which might explain these discrepant results. The future perspective dimension of QLQ‐BR23 (the specific module for breast cancer) was found to be associated with increased risk of death in a study by De Aguiar et al 25.

In clinical practice, as future uncertainty is part of the supplementary HRQoL module QLQ‐BN20, this would theoretically require the submission of both QLQ‐C30 and QLQ‐BN20 questionnaires in their entirety to each patient. This approach might be quite difficult to implement in daily practice, especially for these cognitively impaired patients.

HRQoL baseline data were missing for almost one‐third of the patients in our study. We initially planned to document the reasons for missing baseline HRQoL data. Unfortunately, this parameter is also poorly documented and when available very heterogeneous, leading to the impossibility of providing robust reasons for HRQoL baseline data in the study. It can only be assumed that the lack of response was due to major cognitive dysfunction due to patient's characteristics, as reflected by the missing data population characteristics. Nevertheless, the survival prognosis between the patients with or without any HRQoL baseline data is not significantly different in this study.

Indeed, baseline MMSE score was significantly lower and cognitive impairment significantly more frequent in the patients with missing HRQoL than in patients with available HRQoL data. The high nonresponse rate might be specific to the field of brain research and raises the question of HRQoL self‐assessment feasibility. A functional alternative could be to create a simple specific tool for assessment of the future uncertainty dimension that allows for both auto‐ and hetero‐evaluation and is more applicable to clinical practice.

From a statistical point of view, the assessment of model performance measures, such as discrimination, calibration, and internal validation, strengthen the present investigation. Although the model developed here has good calibration, discrimination and robust internal validation (reproducibility), these results, from an exploratory analysis, must be confirmed in a prospectively recruited validation study to ensure their wider transportability and generalizability. This external validation could allow to confirm the cut‐off values for the HRQoL score.

This study also has some limitations. Indeed, despite the quite homogeneous UGB population analyzed, the sample size of patients may lead to a lack of statistical power to detect other associations. Finally, the score proposed must be improved with other parameters not available in the trial, such as the MGMT status.

In conclusion, this study confirmed the prognostic value of HRQoL in patients with UGB. The assessment of the HRQoL at baseline could guide clinicians in stratifying risk for death in these patients and in providing a basis for early and adapted therapeutic interventions. The determination of HRQoL at baseline should facilitate death risk stratification and might also be useful in optimizing the design of future clinical trials.

## Conflict of Interest

OC received honoraria from Roche as principal investigator of the AVAGLIO trial, which was sponsored by Roche. FB received a grant and honorarium from Roche. All remaining authors have declared no conflicts of interest.

## Supporting information


**Figure S1.** Kaplan–Meier curve of overall survival according to HRQoL availability.
**Table S1.** Distribution of HRQoL scores.
**Table S2.** The PROSUG score.
