# Supplementary material for: Prognostic value of health‐related quality of life for death risk stratification in patients with unresectable glioblastoma
Source: Cancer Med. 2016 Jun 1;5(8):1753–64. doi: 10.1002/cam4.734 (PMC4893352; doi:10.1002/cam4.734)
Supplement: Supplementary file 1 — Figure S1. Kaplan–Meier curve of overall survival according to HRQoL availability. Table S1. Distribution of HRQoL scores. Table S2. The PROSUG score. [file CAM4-5-1753-s001.doc]

**Table S1: Distribution of HRQoL scores**

|  | **N=102** |  | **N** | **Mean** | **SD** | **Median** | **Min.** | **Max.** |
| --- | --- | --- | --- | --- | --- | --- | --- | --- |
| **QLQ-C30** | Functional scores | Global Health Status | 98 | 49.8 | 22.6 | 50.0 | 0.0 | 100.0 |
|  | 0 (worst) to 100 (best) | Physical Functioning | 100 | 74.0 | 27.8 | 80.0 | 0.0 | 100.0 |
|  |  | Role Functioning | 98 | 52.9 | 35.5 | 66.7 | 0.0 | 100.0 |
|  |  | Emotional Functioning | 98 | 66.0 | 25.9 | 66.7 | 0.0 | 100.0 |
|  |  | Cognitive Functioning | 99 | 58.7 | 31.3 | 66.7 | 0.0 | 100.0 |
|  |  | Social Functioning | 97 | 64.8 | 34.4 | 66.7 | 0.0 | 100.0 |
|  | Symptomatic scores | Fatigue | 100 | 37.9 | 25.2 | 33.3 | 0.0 | 100.0 |
|  | 0 (best) to 100 (worst) | Nausea and Vomiting | 100 | 5.5 | 14.2 | 0.0 | 0.0 | 83.3 |
|  |  | Pain | 99 | 24.0 | 30.0 | 16.7 | 0.0 | 100.0 |
|  |  | Dyspnoea | 98 | 10.2 | 18.8 | 0.0 | 0.0 | 66.7 |
|  |  | Insomnia | 95 | 36.8 | 36.5 | 33.3 | 0.0 | 100.0 |
|  |  | Appetite Loss | 96 | 8.3 | 20.5 | 0.0 | 0.0 | 100.0 |
|  |  | Constipation | 97 | 18.6 | 30.4 | 0.0 | 0.0 | 100.0 |
|  |  | Diarrhoea | 99 | 6.1 | 18.0 | 0.0 | 0.0 | 100.0 |
|  |  | Financial difficulties | 96 | 10.1 | 23.3 | 0.0 | 0.0 | 100.0 |
| **BN20** |  |  |  |  |  |  |  |  |
|  |  | Future Uncertainty | 99 | 42.1 | 28.4 | 41.7 | 0.0 | 100.0 |
|  |  | Visual Disorder | 97 | 17.0 | 24.8 | 11.1 | 0.0 | 100.0 |
|  |  | Motor Dysfunction | 98 | 27.1 | 30.2 | 11.1 | 0.0 | 100.0 |
|  |  | Communication Deficit | 99 | 31.7 | 32.1 | 22.2 | 0.0 | 100.0 |
|  |  | Headache | 99 | 23.2 | 29.9 | 0.0 | 0.0 | 100.0 |
|  |  | Seizures | 94 | 1.4 | 10.8 | 0.0 | 0.0 | 100.0 |
|  |  | Drowsiness | 99 | 33.7 | 29.2 | 33.3 | 0.0 | 100.0 |
|  |  | Itchy Skin | 96 | 8.7 | 23.8 | 0.0 | 0.0 | 100.0 |
|  |  | Hair loss | 88 | 1.9 | 10.5 | 0.0 | 0.0 | 66.7 |
|  |  | Weakness of Legs | 96 | 27.4 | 31.7 | 33.3 | 0.0 | 100.0 |
|  |  | Bladder Control | 97 | 16.5 | 29.7 | 0.0 | 0.0 | 100.0 |

**SD: standard deviation**

**Supplementary Figure S1: Kaplan Meier curve of overall survival according to HRQoL availability**

**Supplementary table S2: The PROSUG score**

|  |  |  | **sensitivity deficit** | |
| --- | --- | --- | --- | --- |
|  |  |  | **No** | **Yes** |
|  |  | **Points** | **0** | **1** |
| **Future uncertainty** | **<50** | **0** | 0 | 1 |
|  | **>50** | **1** | 1 | 2 |

|  | **0 point** | **1 point** | **2 points** |
| --- | --- | --- | --- |
|  | **Low-risk** | **Intermediate-risk** | **High-risk** |
| **N (%)** | 51 (52.6) | 39 (40.3) | 7 (7.1) |
| **Median OS (month)** | 16.2 | 9.2 | 4.5 |
| **95% CI** | 13.1 – 19.8 | 6.4 – 14.7 | 1.0 – NA |

- low-risk group: patients with a future uncertainty score<50 without sensitivity deficit (N=52, OS=16.2 months [9.9 – 18.1]);
- intermediate risk group: patients with a future uncertainty score>50 or with sensitivity deficit, (N=40, OS=8.6 months [6.4 – 14.7]);
- high-risk group: patients with a future uncertainty score>50 with sensitivity deficit (N=7, OS=4.5 months [1.0 – NA]), (p-value<0.001).
